# Flow-Mediated Skin Fluorescence Assessment of Microvascular Function in Connective Tissue Diseases: Associations with Nailfold Capillaroscopic Patterns

**DOI:** 10.3390/jcm15145357

**Published:** 2026-07-09

**Authors:** Magdalena Spałkowska, Brygida Marczyk, Jarosław Nowakowski, Krzysztof Batko, Elżbieta Broniatowska, Joanna Kosałka-Węgiel, Beata Kwaśny-Krochin, Anna Wojas-Pelc, Mariusz Korkosz

**Affiliations:** 1 Department of Dermatology, Faculty of Medicine, Jagiellonian University Medical College, Botaniczna 3, 31-503 Kraków, Poland; krzysztofp.batko@doctoral.uj.edu.pl (K.B.); anna.wojas-pelc@uj.edu.pl (A.W.-P.); 2 Jagiellonian Centre for Experimental Therapeutics, Bobrzyńskiego 14, 30-348 Kraków, Poland; brygida.marczyk@jcet.eu; 3 Department of Rheumatology and Immunology, Faculty of Medicine, Jagiellonian University Medical College, Jakubowskiego 2, 30-688 Kraków, Poland; jaroslaw.nowakowski@uj.edu.pl (J.N.); joanna.kosalka@uj.edu.pl (J.K.-W.); beata.kwasny-krochin@uj.edu.pl (B.K.-K.); mariusz.korkosz@uj.edu.pl (M.K.); 4 Department of Rheumatology, Immunology and Internal Medicine, University Hospital in Krakow, Jakubowskiego 2, 30-688 Kraków, Poland; 5 Department of Bioinformatics and Public Health, Faculty of Health Sciences, Andrzej Frycz Modrzewski Krakow University, Herlinga-Grudzińskiego 1, 30-705 Kraków, Poland; ebroniatowska@afm.edu.pl

**Keywords:** Raynaud, systemic sclerosis, microvascular dysfunction, nailfold capillaroscopy, lupus erythematosus, NADH fluorescence

## Abstract

**Background/Objectives:** Microvascular dysfunction is a common early manifestation of connective tissue diseases (CTDs), yet practical non-invasive tools remain limited beyond nailfold capillaroscopy. Flow-mediated skin fluorescence (FMSF), based on changes in nicotinamide adenine dinucleotide (NADH) fluorescence during brachial artery occlusion and reperfusion, is thought to reflect microvascular and mitochondrial function. We investigated whether FMSF differentiates CTD subgroups and how its parameters relate to capillaroscopic findings. **Methods:** In this exploratory cross-sectional study, we examined 99 adults: 19 with lupus erythematosus (LE), 25 with primary Raynaud phenomenon (PR), 29 within the systemic sclerosis (SSc) spectrum, and 26 healthy controls. FMSF parameters were compared across subgroups and capillaroscopic patterns (normal, nonspecific, scleroderma-like) using false discovery rate (FDR) correction and age adjustment. **Results:** Ten of 19 FMSF parameters differed among subgroups (all FDR-corrected *p* ≤ 0.03); after age adjustment, four remained significant (PSD1, endothelial and myogenic oscillations, and hypoxia sensitivity [HS]; all *p* ≤ 0.023), a gradient driven largely by the SSc spectrum. These differences attenuated to non-significance after excluding vasoactive-treated patients (HS *p* = 0.29) and when restricted to an overlapping age range (*p* = 0.06–0.18). Across capillaroscopic patterns, the hyperemic response index and HR max declined toward scleroderma-like patterns (both *p* = 0.007), but no parameter remained associated with pattern after adjustment for CTD diagnosis. **Conclusions:** Our pilot study provides valuable, though exploratory findings: the FMSF signal was largely SSc-driven and sensitive to treatment and age, rather than a robust, disease-independent discriminator. Confirmation in larger, longitudinal cohorts is required.

## 1. Introduction

Cardiovascular disease is a common complication of lupus erythematosus (LE), particularly in its systemic form, systemic lupus erythematosus (SLE). It has been hypothesized that the interplay between traditional cardiovascular risk factors, chronic inflammation, and immune dysregulation contributes to the distinctive cardiovascular risk profile observed in patients with LE [1]. Endothelial dysfunction is an early event in the development of atherosclerosis and is also a hallmark of various forms of vasculopathy. Impaired microvascular function represents an early and defining feature of systemic sclerosis (SSc), often manifesting clinically as Raynaud phenomenon. In advanced stages, patients may develop severe complications, including digital ulcers and amputations. Pulmonary vascular involvement is also common in SSc and may lead to pulmonary arterial hypertension [2]. Similar vascular manifestations have been described in mixed connective tissue disease (MCTD) [3]. An increased risk of cardiovascular disease has been well documented not only in SLE but also in cutaneous lupus erythematosus (CLE) [1,4]. Cardiovascular morbidity in these populations is particularly noteworthy because it frequently affects relatively young individuals and often appears disproportionate to the burden of traditional cardiovascular risk factors. In patients with SLE, the cardiovascular risk has been reported to be comparable to, or even exceed, that observed in individuals with diabetes mellitus [5]. Previous studies have demonstrated the presence of endothelial dysfunction in SLE, even in the absence of clinically overt cardiovascular disease [6]. Moreover, capillaroscopic studies have linked microvascular abnormalities with altered immune activation and increased frequencies of activated lymphocyte subpopulations [7]. Recent developments in cardiovascular risk assessment have highlighted the need for more individualized approaches in clinical populations characterized by inherently elevated cardiovascular risk [8]. Therefore, there remains an unmet need for tailored, non-invasive methods for the assessment of vascular dysfunction in patients with connective tissue diseases (CTDs).

Flow-mediated skin fluorescence (FMSF) is a reproducible, non-invasive technique that provides indirect information on mitochondrial activity and microvascular function by measuring changes in the fluorescence of reduced nicotinamide adenine dinucleotide (NADH) within the skin microcirculation [9]. NADH is a key redox coenzyme involved in cellular energy metabolism. Under normoxic conditions, NADH is oxidized to NAD^+^, whereas ischemia promotes the accumulation of its reduced form [9,10]. The FMSF technique exploits the autofluorescence properties of NADH, which is excited at a wavelength of approximately 340 nm and emits fluorescence at approximately 460 nm. Dynamic changes in the fluorescence signal during ischemia and reperfusion are believed to reflect the functional status of microcirculation, which can be mathematically described as purported to characterize microcirculatory function [11]. Previous studies have demonstrated good reproducibility and preliminary clinical validity of this method [12]. Pilot investigations have explored the utility of FMSF in cardiovascular diseases [13], diabetes mellitus [14], and pulmonary disorders [15]. The technique appears particularly relevant in CTDs, including SLE, SSc, and MCTD, all of which may present with varying degrees of functional and structural vascular abnormalities [1,2,3].

At present, few clinical tools are available for the evaluation of microcirculatory dysfunction beyond nailfold capillaroscopy. Therefore, the aim of this study was to investigate the potential role of FMSF in differentiating patients with various CTDs and clinically relevant subgroups. In addition, we sought to determine the extent to which FMSF-derived parameters correspond to nailfold capillaroscopic findings. Given the limited evidence regarding FMSF in connective tissue diseases, this study was designed as an exploratory, hypothesis-generating investigation.

## 2. Materials and Methods

### 2.1. Study Design and Participants

This cross-sectional study was conducted at the Departments of Dermatology, Rheumatology, Immunology, and Internal Medicine of the University Hospital in Kraków, Poland, a tertiary referral center. Consecutive patients attending outpatient clinics between 2021 and 2022 were screened for eligibility.

Patients were eligible for inclusion in the CTD and primary Raynaud phenomenon (PR) groups if they fulfilled the following criteria: (i) age ≥ 18 years and (ii) presentation with Raynaud phenomenon, either primary or secondary, at the baseline visit. Healthy controls were required to be aged ≥ 18 years and to have no history of Raynaud phenomenon or connective tissue disease.

Systemic lupus erythematosus was classified according to the 2019 American College of Rheumatology/European Alliance of Associations of Rheumatology (ACR/EULAR criteria. Systemic sclerosis (SSc) was classified using the 2013 ACR/EULAR criteria, whereas mixed connective tissue disease was diagnosed according to the Alarcón-Segovia and Villareal criteria. Primary and secondary Raynaud phenomenon were defined using established consensus criteria, supported by nailfold capillaroscopy when clinically indicated. Although antinuclear antibodies (ANA) positivity and nonspecific capillaroscopic findings were observed in a subset of participants, none fulfilled classification criteria for CTD at the time of inclusion and during 4 years of follow-up. Cases of cutaneous lupus erythematosus (CLE) were confirmed histopathologically by skin biopsy.

The exclusion criteria were as follows: (i) clinically manifest atherosclerotic cardiovascular disease, including both central and peripheral vascular disease; (ii) diabetes mellitus, regardless of subtype; (iii) pregnancy; (iv) the postpartum period; and (v) active malignancy.

The study protocol was approved by the local Bioethics Committee (Decision No. 1072.6120.104.2021; approval date: 19 May 2021). Written informed consent was obtained from all participants before enrollment.

### 2.2. Clinical Subgroups

Four clinical subgroups were defined a priori. Healthy volunteers and individuals with idiopathic (primary) Raynaud phenomenon were analyzed separately as non-CTD reference groups. Patients with isolated cutaneous lupus erythematosus and those with systemic lupus erythematosus were combined into a single lupus erythematosus (LE) group.

Patients fulfilling the 2013 ACR/EULAR classification criteria for systemic sclerosis, together with patients with mixed connective tissue disease and scleroderma-overlap syndromes who exhibited skin thickening of the fingers of both hands extending proximal to the metacarpophalangeal joints, were analyzed as a single “SSc spectrum” group. This approach was based on the presence of a shared scleroderma phenotype characterized by clinically overt fibrotic and microvascular involvement, rather than on the underlying diagnostic label alone.

### 2.3. Flow-Mediated Skin Fluorescence Assessment

Flow-mediated skin fluorescence is a novel, non-invasive technique designed to quantitatively assess microvascular function and in vivo cellular metabolism. Measurements were performed using the validated AngioExpert device (Angionica Ltd., Lodz, Poland). The method assumes that changes in skin autofluorescence can be detected in response to transient ischemia and reperfusion induced by brachial artery occlusion. Several FMSF-derived parameters have been proposed to characterize endothelial function, tissue oxygenation, and mitochondrial activity (see Supplementary Table S1).

NADH fluorescence is measured at an emission wavelength of 460 nm following excitation at 340 nm. Due to the limited penetration depth of the excitation beam, most of the signal originated from the epidermis and papillary dermis. These skin layers have relatively low vascular density; therefore, changes in NADH fluorescence primarily reflect oxygen delivery from deeper vascular structures.

The measurement protocol evaluates three physiological domains:

1. Baseline flowmotion under resting (normoxic) conditions;
2. The ischemic response during forearm occlusion;
3. The reactive hyperemic response following cuff release.

A detailed description of the measurement technique and parameter calculations has been published previously [16]. High reproducibility and measurement agreement have been reported for this method [12,17].

Assessments were performed in standardized conditions by two experienced, unblinded FMSF investigators. Subjects were asked to remain seated during the study visit, with a 10-minute rest period in a dedicated examination room with air conditioning. For 3 min, baseline was recorded; subsequently, the brachial artery was pressurized using tailored cuff inflation and constriction at over 60 mmHg systolic pressure, with monitoring of the ischemic response for 3 min. After occlusion for 3 min, the cuff was released, with observation of the reperfusion response lasting 4 min. Throughout the examination, NADH fluorescence of the forearm epidermis was registered for 10 min.

### 2.4. Nailfold Capillaroscopy

Nailfold capillaroscopy was performed in all participants on eight fingers of both hands, excluding the thumbs. Assessments were conducted independently by two experienced investigators: a rheumatologist using a Dino-Lite digital capillaroscope at 50× as well as 200× magnification, and a dermatologist–immunologist using Dino-Lite digital capillaroscopy at 500× magnification in conjunction with a Heine Delta dermoscope (10× magnification). The evaluators were aware of the participants’ clinical diagnoses, but assessed the capillaroscopic findings independently; no disagreements occurred. Capillaroscopic patterns were classified as normal, nonspecific, or scleroderma-like according to established criteria.

### 2.5. General Assessment

Current laboratory results were reviewed at the qualification visit, including metabolic parameters, complete blood count, complement components (C3, C4), and autoimmune serology: antinuclear antibodies (ANA), anti-double-stranded DNA (anti-dsDNA), and antiphospholipid antibodies (aPL). In all patients with CLE, SLE, MCTD, and SSc, we evaluated the level of systemic involvement of the disease. Joint involvement was confirmed in ultrasound, laboratory, and clinical assessment. In symptomatic patients, pulmonary involvement was confirmed by high-resolution computed tomography, echocardiography, and diffusing capacity of the lung for carbon monoxide (DLCO) assessment, with the DETECT algorithm applied for pulmonary arterial hypertension screening in SSc. Esophageal dysmotility was assessed by high-resolution manometry. Cytopenia was defined as the presence of at least one of the following: anemia, leukopenia or lymphopenia, or thrombocytopenia, according to standard laboratory reference ranges. In all patients, we collected data concerning the comorbidities, e.g., the presence of other autoimmune diseases such as Sjögren’s syndrome.

### 2.6. Statistical Analysis

Statistical analyses were performed using R software version 4.5.3 (R Core Team, 2026; R Foundation for Statistical Computing, Vienna, Austria). Continuous variables are presented as median and interquartile range (IQR), whereas categorical variables are reported as frequencies and percentages (N, %).

Prior to analysis, five participants with implausible FMSF values attributable to measurement artifacts were excluded. In addition, one control participant was retrospectively excluded after a diagnosis of rheumatoid arthritis had been established in the follow-up period.

FMSF parameters were compared across the predefined clinical subgroups (Controls, LE, PR, SSc) and according to capillaroscopic patterns (normal, nonspecific, and scleroderma-like) using the Kruskal–Wallis test, with ε^2^ reported as a measure of effect size. Age-adjusted comparisons were performed using rank-based analysis of covariance (ANCOVA), with partial η^2^ used to quantify effect size. We consider age-adjusted analyses as the main comparisons for inferential interpretation.

When the Kruskal–Wallis test indicated statistically significant differences, pairwise comparisons were conducted using the Wilcoxon rank-sum test. We used the Hodges–Lehmann estimator for between-group location shifts to report median differences, with 95% confidence intervals (CIs) and rank-biserial coefficients (unadjusted, exploratory).

Trends across capillaroscopic categories were evaluated using the Jonckheere-Terpstra permutation test (5000 replicates). Categorical variables were compared using Fisher’s exact test with Monte Carlo simulation (100,000 replicates), and Cramér’s V was reported as the corresponding measure of effect size.

Associations between age and FMSF parameters were assessed using Spearman’s rank correlation coefficients, both overall and after adjustment for CTD subgroup. To account for multiple testing, false discovery rate correction according to the Benjamini–Hochberg procedure was applied to the family of FMSF parameters (n = 19). Redundant or collinear variables were excluded a priori.

Finally, exploratory principal component analysis was performed using an imputed dataset generated with the mice package and a reduced set of log-transformed FMSF parameters (n = 13). Given the exploratory nature of the study and the absence of a predefined primary endpoint, the results of the principal component analysis were intended primarily for visualization and hypothesis generation rather than diagnostic classification. To further inform on the robustness of our analyses, we performed sensitivity analyses with subgroups: (i) after exclusion of patients treated with vasoactive agents, and (ii) after excluding patients with cutaneous lupus erythematosus.

All statistical tests were two-sided, and a *p*-value < 0.05 was considered statistically significant. Collinearity among covariates was verified using the generalized variance inflation factor (maximum ≤ 1.32).

## 3. Results

### 3.1. Demographic and Clinical Characteristics of the Study Cohort

The final analytical sample comprised 29 patients within the SSc spectrum, 19 patients with LE, 25 patients with PR, and 26 healthy controls (see flowchart Figure S1). The SSc spectrum group included patients fulfilling the classification criteria for systemic sclerosis, as well as individuals with mixed connective tissue disease and scleroderma-overlap syndromes who exhibited a scleroderma phenotype characterized by skin thickening of the fingers extending proximal to the metacarpophalangeal joints. Demographic and baseline characteristics are summarized in Table 1.

Clinical subgroups differed substantially in age distribution (*p* < 0.001). The median (IQR) age was 30.0 (IQR 23.0–43.0) years in PR, which characterizes these patients as the youngest. Conversely, patients with SSc were the oldest, with a median (IQR) age of 56.0 (IQR 47.0–66.0) years. Healthy controls and LE patients were middle-aged in general, with median (IQR) of 40.0 (IQR 33.0–52.0) years and 55.0 (45.5–61.5) years, respectively.

Body mass index also differed across the study groups (*p* = 0.009), with the lowest values observed among patients with PR. Female participants predominated in all subgroups, accounting for 100% of the LE and PR cohorts, 89.7% of the SSc spectrum cohort, and 65.4% of healthy controls.

The distribution of immunological markers was consistent with the underlying clinical diagnoses. Antinuclear antibody positivity was observed in all patients within the SSc spectrum and in 94.7% of patients with LE, compared with 44.0% of patients with PR and 7.7% of healthy controls (*p* < 0.001).

### 3.2. Comparison of Capillaroscopic Features Across Clinical Subgroups

**Figure 1:**
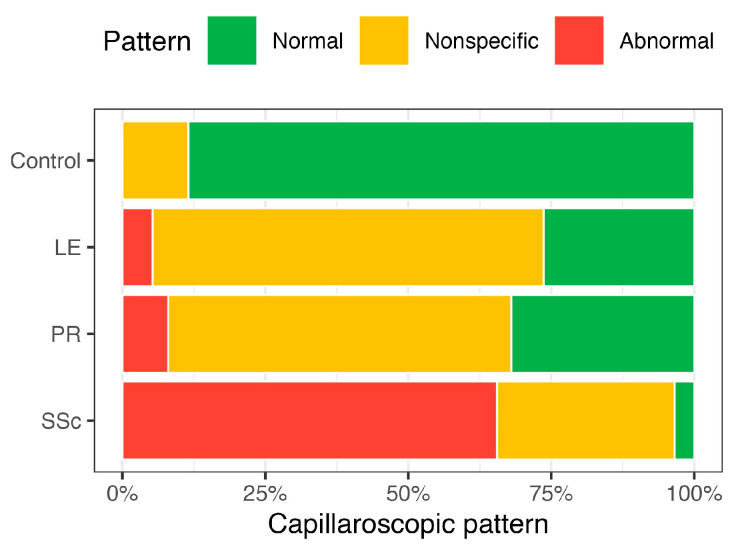
Distribution of nailfold capillaroscopic patterns across the clinical subgroups. Abbreviations: lupus erythematosus, LE; primary Raynaud phenomenon, PR; systemic sclerosis spectrum, SSc.

Capillaroscopic patterns differed significantly among the clinical subgroups (Figure 1; Fisher’s exact *p* < 0.001; Cramér’s V = 0.62).

A normal capillaroscopic pattern was observed predominantly in healthy controls (88.5%) but was considerably less frequent in patients with PR (32.0%) and LE (26.3%), and was rare in the SSc spectrum group (3.4%). In contrast, a scleroderma-like pattern was present in most patients within the SSc spectrum (65.5%), whereas it was absent in healthy controls and uncommon in the PR (8.0%) and LE (5.3%) groups. A nonspecific pattern represented the most frequent finding in patients with LE (68.4%) and PR (60.0%).

**Figure 2:**
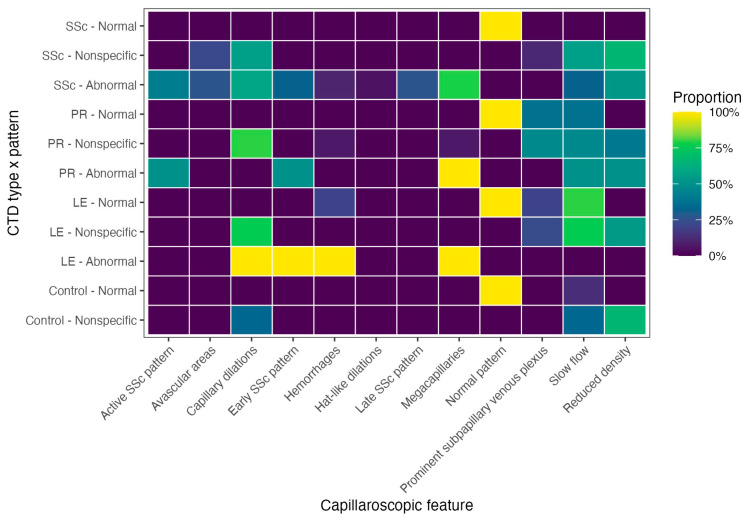
Frequency of individual nailfold capillaroscopic abnormalities according to clinical subgroup. Abbreviations: lupus erythematosus, LE; primary Raynaud phenomenon, PR; systemic sclerosis spectrum, SSc.

The distribution of individual capillaroscopic abnormalities was similarly consistent with the expected disease phenotypes (Figure 2). Megacapillaries, capillary dilatation, and reduced capillary density were most frequently observed in patients within the SSc spectrum (51.7%, 55.2%, and 55.2%, respectively). Early, active, and late scleroderma patterns were identified almost exclusively in this group, occurring in 20.7%, 27.6%, and 17.2% of patients, respectively. Slow capillary flow was most common among patients with LE (73.7%), whereas a prominent subpapillary venous plexus was observed most frequently in the PR group (40.0%).

### 3.3. Comparison of Flow-Mediated Skin Fluorescence Parameters Across Clinical Subgroups

A comparison of FMSF parameters across the clinical subgroups is presented in Table 2.

Ten of nineteen evaluated FMSF parameters differed significantly among the clinical subgroups after correction for multiple testing (all FDR-adjusted *p* ≤ 0.03). The largest between-group differences were observed for hypoxia sensitivity (HS), PSD1, myogenic oscillations, and endothelial oscillations (all FDR-adjusted *p* ≤ 0.003). Additionally, reactive myogenic %, PSD2, hyperemic response index, neurogenic oscillations, reactive neurogenic %, and reactive endothelial % were also significant following FDR correction.

Because the clinical subgroups differed substantially in age, additional analyses were performed using rank-based ANCOVA adjusted for age. Following age adjustment, only four parameters remained statistically significant after FDR correction: PSD1, endothelial oscillations, myogenic oscillations, and HS (all FDR-adjusted *p* ≤ 0.023); these differences persisted after additional adjustment for sex, BMI, dyslipidemia, and hypertension (see Supplementary Table S2).

Pairwise comparisons of HS revealed significantly lower values in patients with LE compared with those with PR, patients with LE were characterized by lower (median shift of −58.3, 95% CI −112.7 to −31.7; rank-biserial correlation = −0.73; *p* < 0.001). Similarly, HS values were lower in patients within the SSc spectrum than in those with PR (median shift −55.7, 95% CI −97.1 to −30.6; rank-biserial correlation = −0.65; *p* < 0.001).

Healthy controls also exhibited higher HS values than patients with LE (median shift: 31.1, 95% CI 6.2 to 64.6; r = 0.46; *p* = 0.017) and those within the SSc spectrum (median shift 29.0, 95% CI 4.6 to 54.2; r = 0.40; *p* = 0.017), whereas no significant difference was observed between healthy controls and patients with PR (r = −0.29, *p* = 0.090). Likewise, no significant differences in HS were detected between the LE and SSc groups (r = −0.05, *p* = 0.80).

A similar pattern was observed for PSD1, with higher values in healthy controls and patients with PR than in patients with LE and those within the SSc spectrum. The most pronounced differences were observed between LE and PR (median shift −49.8, 95% CI −85.0 to −19.0; rank-biserial correlation = −0.59; *p* = 0.002) and between PR and the SSc spectrum group (median shift 52.0, 95% CI 21.0 to 78.5; rank-biserial correlation = 0.56; *p* = 0.001).

In additional sensitivity analyses, we observed robust evidence for PSD1, endothelial, and myogenic oscillations, as well as HS, to be significantly different across clinical subgroups (even after FDR correction). This was true for restriction to SLE alone (all FDR corrected *p* values < 0.05; see Supplementary Table S3), including consistently lower HS in PR, as compared with healthy controls (rank-biserial −0.70 and −0.42, respectively). After excluding 26 patients receiving vasoactive agents, we observed comparable effect sizes (partial n2 range 0.09–0.15), but also lower HS in LE, as compared to PR (rank-biserial −0.69, *p* = 0.001). However, the age-adjusted comparison based on the omnibus test was no longer significant, which may reflect reduced sample size.

### 3.4. Comparison of Flow-Mediated Skin Fluorescence Parameters Across Capillaroscopic Patterns

Comparisons of FMSF parameters according to capillaroscopic pattern are summarized in Table 3. 

Five FMSF parameters differed significantly across capillaroscopic categories after FDR correction (HRI, *p* = 0.003), HR max (*p* = 0.005), PSD1 (*p* = 0.010), myogenic oscillations (*p* = 0.011), and RHR (*p* = 0.015). 

Except for myogenic oscillations, these parameters demonstrated a progressive decline from normal to nonspecific and subsequently to scleroderma-like patterns. Myogenic oscillations were comparable in the normal and nonspecific groups (median values: 14.9 vs. 15.2) but were substantially lower in participants with a scleroderma-like pattern (median 3.9).

After age adjustment, across capillaroscopic patterns, only HRI (*p* = 0.007), HR max (*p* = 0.007), and RHR (*p* = 0.049) were significant. Trend tests across the ordered pattern support monotonic associations for HRI and HR max (both trend *p* = 0.004), PSD1, myogenic oscillations, and RHR (all trend *p* = 0.005), as well as endothelial oscillations (trend *p* = 0.014). Reactive endothelial % was the only parameter to increase monotonically across patterns (*p* = 0.02 unadjusted).

At the individual level, single FMSF parameters discriminated a scleroderma-like pattern only modestly (best AUROC ≈ 0.72–0.74) and added little beyond age (see Supplementary Table S4).

### 3.5. Relationship Between Age and Flow-Mediated Skin Fluorescence Parameters

Monotonic associations between age and each FMSF parameter are presented in Table 4. In the pooled Spearman correlation analysis, HS showed the strongest association with age (rho = −0.47, *p* < 0.001), reactive myogenic % decreased with age (rho = −0.42, *p* < 0.001), and reactive endothelial % increased with age (rho = 0.39, *p* < 0.001). PSD1, as well as myogenic and neurogenic oscillations, also showed moderate decreases with increasing age *(p* ≤ 0.002). Overall, nine of the 19 FMSF parameters remained significantly associated with age in the pooled analysis after false discovery rate (FDR) correction. However, after adjustment for the CTD subgroup using partial Spearman correlation, most of these associations were attenuated.

### 3.6. Principal Component Analysis of Flow-Mediated Skin Fluorescence Parameters

**Figure 3:**
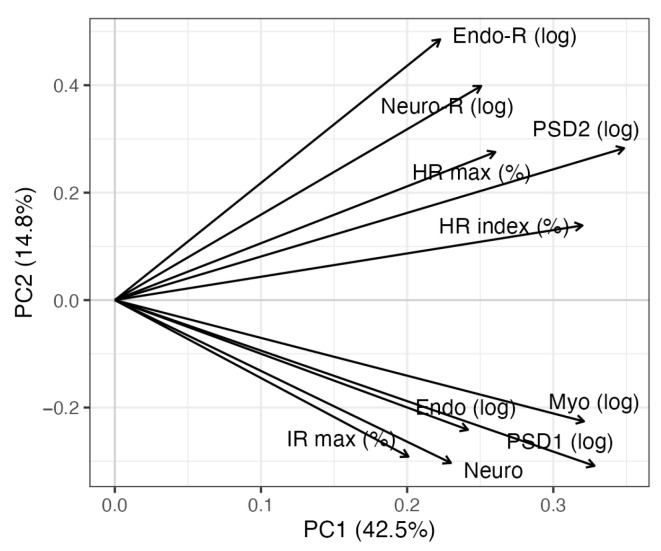
Score plot from the principal component analysis of flow-mediated skin fluorescence parameters comparing the clinical subgroups.

**Figure 4:**
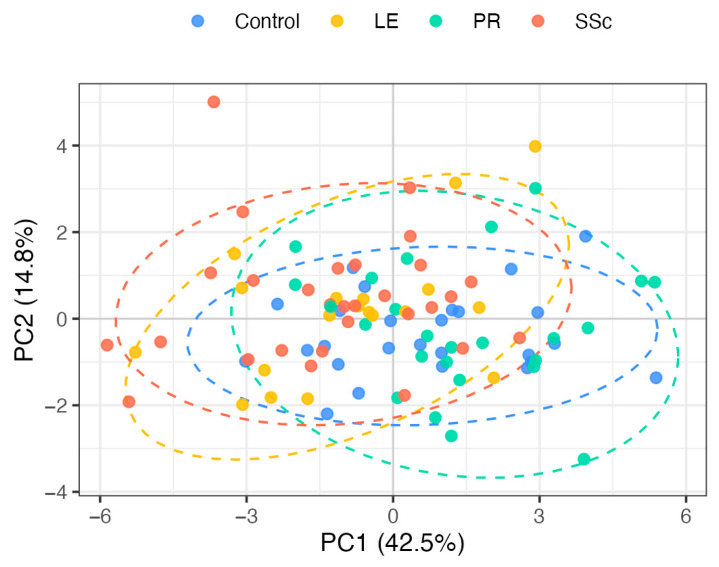
Loading plot based on principal component analysis of flow-mediated skin fluorescence features.

Principal component analysis (PCA) of the FMSF parameters identified two principal components that together explained 57.3% of the total variance (PC1 = 42.5%, PC2 = 14.8%; Figure 3). Overall, PC1 could be interpreted as a proxy for microvascular activity, based on strong links with PSD2, PSD1, myogenic oscillations, HR index, and HR max (Figure 4). In contrast, PC2 differentiated baseline from reactive measurements, with one end of the spectrum characterized by reactive endothelial and reactive neurogenic oscillations and the other by baseline parameters (PSD1, myogenic, neurogenic, endothelial, and IR max).

Healthy controls and PR patients tended to cluster separately from LE and SSc patients along PC1, consistent with the higher overall microvascular activity observed in the former groups. However, substantial overlap between subgroups was evident for both PC1 and PC2.

## 4. Discussion

This exploratory study identified differences in FMSF parameters across CTD subgroups, primary Raynaud phenomenon, and healthy controls, as well as across capillaroscopic patterns. After adjustment for multiple testing and age, four parameters (PSD1, endothelial oscillations, myogenic oscillations, and hypoxia sensitivity [HS]) remained significantly different among the clinical subgroups, with higher values in healthy controls and patients with PR and lower values in patients with LE and within the SSc spectrum. However, the gradient is mainly derived from the contrast with the SSc spectrum. All four age-adjusted differences were observed to be attenuated when vasoactive drugs were excluded and when comparisons were restricted to the overlapping age range. Particularly, HS appears to be strongly tied to patients’ age, with the PR to control difference insignificant after adjustment.

Across capillaroscopic patterns, the hyperemic parameters HRI, HR max, and RHR remained associated with capillaroscopic abnormality after age adjustment, with a progressive decline from normal to nonspecific to scleroderma-like patterns. However, these associations were not independent of the underlying disease: after additional adjustment for CTD diagnosis, no FMSF parameter remained associated with capillaroscopic pattern (see Supplementary Table S5). Therefore, the FMSF–capillaroscopy relationship appears to be largely explained by shared disease (predominantly SSc) rather than reflecting a capillaroscopy-specific signal. Taken together and given the substantial between-group overlap evident on principal component analysis, our findings merit further study: FMSF may track microvascular functional differences across clinical CTD subgroups, but it did not facilitate independent cross-group differentiation. Our findings show that changes in FMSF parameters are only partially consistent with morphological pathology in the nailfold, thus suggesting that both methods could be complementary to one another (in terms of microvascular function assessment).

FMSF is a relatively recently developed, non-invasive technique for the assessment of cutaneous vascular responses and has been increasingly investigated in a range of clinical settings [13]. Several FMSF-derived parameters have been proposed to reflect aspects of microvascular function and cellular metabolism within the skin, although their precise physiological interpretation in vivo remains incompletely understood. Because vascular responses in the dermis may capture broader features of systemic vascular dysfunction, our findings support the presence of altered microvascular responses across the CTD groups studied [12,18,19].

The pronounced abnormalities observed in patients within the SSc spectrum are consistent with the well-established burden of vasculopathy associated with systemic sclerosis and provide support for the biological relevance of the FMSF findings in this population. Interestingly, patients with LE exhibited an FMSF profile that was broadly comparable to that observed in the SSc spectrum, despite the markedly lower prevalence of scleroderma-like capillaroscopic abnormalities. This discordance suggests that functional alterations detected by FMSF are not fully concordant with morphological findings on nailfold capillaroscopy and that these techniques may capture complementary aspects of microvascular involvement. However, given the cross-sectional design of the present study, no conclusions can be drawn regarding the temporal relationship between functional abnormalities detected by FMSF and structural changes identified by capillaroscopy. Longitudinal studies are required to determine whether these alterations evolve in parallel or represent different stages of microvascular dysfunction. There are several potential explanations for microvascular impairment in lupus erythematosus, which include endothelial dysfunction, effects of immune complex-mediated and interferon type-I associated damage, and accelerated development of CV risk factors; all of which, together, may shape a different vasculopathy profile to overt scleroderma-like remodeling of capillaries.

From a mechanistic perspective, the NAD+/NADH pathway plays a central role in cellular energy metabolism and has been implicated in the regulation of inflammatory responses, oxidative stress, and mitochondrial homeostasis [20]. Nicotinamide, a precursor of NAD+, has demonstrated both anti-inflammatory and vasoprotective effects in preclinical models [21,22]. These observations provide a biologically plausible framework for interpreting alterations in FMSF signals in disease states characterized by chronic inflammation and vascular dysfunction.

Previous studies have applied FMSF in a variety of clinical settings to investigate abnormalities in vascular responses. In untreated hypertension, an exaggerated increase in skin fluorescence during the early ischemic phase was interpreted as a potential marker of altered mitochondrial function [23]. In a small case–control study including patients with SLE and healthy controls, reduced ischemic and hyperemic responses were reported, suggesting impaired endothelial reactivity [24]. In that study, FMSF-derived parameters were negatively associated with age but not with disease activity or the extent of organ involvement [24]. Similar alterations in ischemic and hyperemic responses have been described in patients with coronary artery disease and diabetes mellitus [12,17], sleep apnea, and pulmonary disorders [15]. In addition, selected FMSF parameters have been associated with circulating markers of endothelial injury [17,25].

Our findings extend these observations by demonstrating group-level differences in FMSF-derived responses across the CTD spectrum. The most pronounced differences were observed in baseline oscillatory parameters rather than in reactive indices, suggesting that resting microvascular dynamics may be more sensitive to disease-related alterations than responses to ischemic challenge. However, given the overlap observed between clinical groups and the exploratory nature of the present study, these findings should be considered hypothesis-generating and require confirmation in larger, independent cohorts before any conclusions regarding their diagnostic utility can be drawn.

Age is an important factor to consider when interpreting FMSF parameters, consistent with earlier reports in patients with SLE [24]. In our cohort, nine of the 19 evaluated FMSF parameters were significantly associated with age after FDR correction. Hypoxia sensitivity exhibited the strongest inverse association with age (rho = −0.47), whereas reactive myogenic percentage decreased and reactive endothelial percentage increased with advancing age. Importantly, after age adjustment using rank-based ANCOVA, four FMSF parameters remained significantly different across the clinical subgroups, and three remained significantly associated with capillaroscopic patterns. These findings suggest that the observed differences between subgroups cannot be fully explained by age alone, although residual confounding by age cannot be excluded in the context of a cross-sectional study.

Several limitations of this study should be acknowledged. First, the cross-sectional design precludes causal inference and does not allow conclusions regarding the temporal relationship between functional and structural microvascular abnormalities. Second, the relatively small sample size may have reduced statistical power and limited the generalizability of the findings. Third, the interpretation of FMSF signals in vivo remains, to some extent, assumption-dependent. It is still uncertain to what extent the detected fluorescence signal reflects free versus protein-bound NADH [24], and whether alterations in FMSF directly parallel microvascular and mitochondrial dysfunction requires further experimental validation. Endothelial, neurogenic, and myogenic bands are recorded analogously to laser-Doppler flowmotion and should be interpreted as putative. Potential confounding by medication use should also be considered, as vasoactive and immunomodulatory therapies differed substantially between the clinical groups. Although the effects of most antihypertensive agents on FMSF measurements are thought to be limited, with the possible exception of metoprolol [26], their influence cannot be completely excluded. We were unable to assess the effects of smoking status due to a lack of consistent and reliable recording, which is another unmeasured confounder. However, in sensitivity analyses, excluding patients on vasoactive drugs showed that the magnitude and direction of differences are consistent.

Another limitation is that the investigators performing the FMSF measurements were aware of the participants’ clinical status. Although this may have introduced observer bias, the examinations were conducted by two experienced researchers according to a standardized protocol, and the FMSF output consisted of automated numerical parameters generated by the device rather than subjective assessments. Therefore, the potential influence of observer expectations on the results was likely limited.

Similarly, although the capillaroscopic evaluators were not blinded to the participants’ clinical diagnoses, assessments were performed independently by two experienced clinicians. The observed capillaroscopic abnormalities were generally clear-cut, allowing consistent classification of capillaroscopic patterns. It is also necessary to state that digital capillaroscopy/dermatoscopy, while performed at up to 500× magnification, is not fully comparable with nailfold video capillaroscopy, in that it may be less sensitive to mild microvascular changes.

Importantly, FMSF measurements were obtained from the forearm skin and therefore reflect a generalized cutaneous microvascular bed rather than the nailfold microcirculation directly assessed by capillaroscopy. Consequently, concordance between these methods should be interpreted as indirect rather than representing anatomical equivalence. Additionally, the observed relationships between FMSF parameters and capillaroscopic features are not independent of the underlying primary disorders after adjustment for CTD diagnosis; no FMSF parameter was significant (see Supplementary Table S5). We consider our findings as indicative of CTD phenotype characteristics, rather than capillaroscopy pattern-specific. The sensitivity analyses with the exclusion of patients with artifact measurements showed comparable differences and resulted in the same significant parameter set being differentiated. Larger, longitudinal, and preferably multicenter studies are required to confirm our findings across different CTDs, evaluate their reproducibility, and determine their potential prognostic relevance for clinically meaningful outcomes.

## 5. Conclusions

FMSF represents a candidate for further evaluation as a non-invasive clinical tool for the assessment of microvascular function in connective tissue diseases. In this exploratory cross-sectional study, we identified differences in FMSF-derived functional response across CTD subgroups. Although substantial overlap between groups was observed, the most pronounced capillaroscopic abnormalities in patients within the SSc spectrum were associated with distinct FMSF profiles.

The relationship between FMSF measurements obtained from forearm skin and nailfold capillaroscopic findings should be interpreted as indirect, reflecting potentially complementary aspects of microvascular dysfunction rather than anatomical concordance. Further studies are warranted to validate these observations, establish the clinical utility of FMSF, and determine whether FMSF-derived abnormalities precede the development of overt capillaroscopic changes.

## Figures and Tables

**Table 1:** Demographic and clinical characteristics of the study participants according to clinical subgroup.

Variable	Control (n = 26)	LE (n = 19)	PR (n = 25)	SSc (n = 29)	*p*
Demographics					
Age, years	40.0 (33.0–52.0)	55.0 (45.5–61.5)	30.0 (23.0–43.0)	56.0 (47.0–66.0)	<0.001
BMI, kg/m^2^	24.0 (22.0–25.8)	23.0 (20.5–26.0)	21.0 (20.0–23.0)	25.0 (21.0–28.0)	0.009
Female sex	17 (65.4)	19 (100.0)	25 (100.0)	26 (89.7)	<0.001
Cutaneous lupus subtype *					
Acute cutaneous LE	0 (0.0)	1 (5.3)	0 (0.0)	0 (0.0)	0.19
Subacute cutaneous LE	0 (0.0)	3 (15.8)	0 (0.0)	0 (0.0)	0.006
Discoid LE	0 (0.0)	1 (5.3)	0 (0.0)	1 (3.4)	0.56
Immunology					
ANA positive	2 (7.7)	18 (94.7)	11 (44.0)	27 (100.0)	<0.001
Anti-dsDNA antibody	0 (0.0)	7 (36.8)	0 (0.0)	2 (6.9)	<0.001
Antiphospholipid antibody	0 (0.0)	4 (21.1)	1 (4.0)	3 (10.3)	0.05
Low C3 or C4	1 (3.8)	6 (31.6)	1 (4.0)	4 (13.8)	0.02
Organ involvement					
Oesophageal involvement	0 (0.0)	0 (0.0)	0 (0.0)	11 (37.9)	<0.001
Pulmonary involvement	0 (0.0)	2 (10.5)	0 (0.0)	16 (55.2)	<0.001
Pleural involvement	0 (0.0)	0 (0.0)	0 (0.0)	1 (3.4)	>0.99
Pericardial involvement	0 (0.0)	0 (0.0)	0 (0.0)	4 (13.8)	0.01
Telangiectasias	0 (0.0)	1 (5.3)	0 (0.0)	4 (13.8)	0.07
Arthritis and/or arthralgia	1 (3.8)	7 (36.8)	4 (16.0)	8 (27.6)	0.02
Digital ulcers	0 (0.0)	0 (0.0)	0 (0.0)	7 (24.1)	<0.001
Comorbidities					
Hypertension	1 (3.8)	1 (5.3)	1 (4.0)	4 (13.8)	0.51
Dyslipidemia	10 (38.5)	4 (22.2)	0 (0.0)	2 (6.9)	<0.001
Hypothyroidism	0 (0.0)	1 (5.3)	1 (4.0)	7 (24.1)	0.007
Cytopenia	0 (0.0)	4 (21.1)	2 (8.3)	9 (31.0)	0.004
Medications					
Vasoactive therapy	0 (0.0)	7 (36.8)	4 (16.0)	15 (51.7)	<0.001
Hydroxychloroquine	0 (0.0)	8 (42.1)	0 (0.0)	10 (34.5)	<0.001
Low-dose aspirin	0 (0.0)	0 (0.0)	1 (4.0)	1 (3.4)	0.85
MTX or MMF	0 (0.0)	5 (26.3)	0 (0.0)	12 (41.4)	<0.001
Glucocorticoids	0 (0.0)	8 (42.1)	1 (4.0)	7 (24.1)	<0.001
Capillaroscopic pattern					
Normal	23 (88.5)	5 (26.3)	8 (32.0)	1 (3.4)	<0.001
Nonspecific	3 (11.5)	13 (68.4)	15 (60.0)	9 (31.0)	<0.001
Scleroderma-like	0 (0.0)	1 (5.3)	2 (8.0)	19 (65.5)	<0.001

Abbreviations: ANA, antinuclear antibody; BMI, body mass index; C3/C4, complement components 3 and 4; dsDNA, double-stranded DNA; LE, lupus erythematosus; MMF, mycophenolate mofetil; MTX, methotrexate; PR, primary Raynaud phenomenon; SSc, systemic sclerosis spectrum. * One cutaneous-lupus patient presented with both acute and subacute cutaneous lupus, so the 1 acute, 3 subacute, and 1 discoid lesion counts in the LE column correspond to the four cutaneous-only lupus (CLE) cases. One additional discoid lesion occurred in an SSc-overlap patient.

**Table 2:** Comparison of flow-mediated skin fluorescence parameters across the clinical subgroups before and after age adjustment.

Parameter	Control (n = 26)	LE (n = 19)	PR (n = 25)	SSc (n = 29)	ε^2^	*p* (KW)	FDR *p* (KW)	Partial η^2^	*p* (ANCOVA)	FDR *p* (ANCOVA)
Hypoxia sensitivity (HS)	55.8 (19.9–114.8)	24.5 (11.1–36.2)	90.1 (55.5–213.5)	20.5 (10.6–44.7)	0.26	<0.001	<0.001	0.13	0.005	0.02
PSD1 (baseline flowmotion power)	78.4 (56.7–124.9)	25.0 (15.1–62.0)	84.1 (58.0–154.5)	37.5 (15.6–65.3)	0.24	<0.001	<0.001	0.15	0.001	0.02
Myogenic oscillations	20.8 (7.4–29.1)	5.3 (1.7–15.8)	20.5 (12.1–47.0)	4.0 (2.1–15.5)	0.22	<0.001	<0.001	0.14	0.003	0.02
Endothelial oscillations	34.4 (19.0–49.8)	11.6 (6.3–19.3)	23.3 (13.0–36.1)	12.3 (4.8–25.0)	0.18	<0.001	0.003	0.14	0.002	0.02
Neurogenic oscillations	20.7 (10.5–45.8)	10.6 (4.4–27.5)	33.0 (11.5–55.6)	10.8 (4.1–22.6)	0.13	0.006	0.01	0.05	0.19	0.28
PSD2 (reactive flowmotion power)	100.6 (51.3–196.4)	48.0 (35.0–92.9)	130.2 (89.3–305.6)	79.3 (37.0–122.7)	0.15	0.002	0.006	0.09	0.04	0.11
Reactive endothelial oscillations	20.4 (13.9–36.5)	19.4 (8.6–32.7)	27.1 (15.6–49.0)	25.9 (16.1–42.5)	0.03	0.46	0.51	0.02	0.52	0.58
Reactive neurogenic oscillations	11.8 (8.5–21.0)	9.9 (6.8–13.9)	12.9 (8.8–27.0)	12.6 (5.5–20.7)	0.02	0.67	0.67	0.01	0.78	0.78
HR index (%)	13.1 (10.2–16.5)	12.1 (9.4–14.8)	12.6 (10.9–15.7)	9.3 (8.2–11.9)	0.13	0.006	0.01	0.10	0.02	0.09
HR max (%)	20.6 (17.1–23.6)	20.4 (16.4–23.1)	19.4 (18.0–22.1)	17.4 (14.5–20.4)	0.06	0.11	0.15	0.05	0.18	0.28
RHR (%)	28.5 (26.5–38.0)	27.8 (22.6–32.9)	34.6 (27.0–37.8)	27.1 (20.8–32.1)	0.08	0.06	0.10	0.05	0.19	0.28
MR (%)	92.4 (82.6–101.1)	97.1 (85.3–106.3)	95.3 (82.7–102.7)	84.4 (72.8–97.4)	0.06	0.10	0.15	0.05	0.17	0.28
IR max (%)	11.4 (7.3–17.7)	11.1 (6.9–17.5)	14.1 (10.2–18.5)	12.1 (5.4–17.6)	0.02	0.66	0.67	0.02	0.76	0.78
Endothelial (%)	44.8 (33.3–62.0)	38.8 (18.6–51.8)	28.1 (16.4–47.1)	35.7 (25.5–52.2)	0.06	0.13	0.18	0.05	0.19	0.28
Neurogenic (%)	31.5 (19.4–48.2)	41.0 (24.7–47.1)	36.8 (20.0–48.7)	38.4 (28.1–52.8)	0.03	0.40	0.48	0.05	0.21	0.29
Myogenic (%)	18.8 (11.9–32.8)	21.2 (11.6–37.2)	27.7 (14.7–41.4)	20.2 (6.2–31.9)	0.04	0.24	0.30	0.03	0.35	0.45
Reactive endothelial (%)	21.7 (13.4–44.4)	36.5 (20.2–50.5)	19.0 (10.4–29.9)	39.4 (26.1–51.2)	0.11	0.02	0.03	0.03	0.45	0.54
Reactive neurogenic (%)	15.6 (9.2–23.1)	21.9 (13.2–31.5)	11.8 (6.0–16.2)	19.3 (11.5–28.8)	0.12	0.008	0.02	0.07	0.09	0.23
Reactive myogenic (%)	56.2 (35.7–77.5)	37.8 (24.8–64.4)	66.0 (57.1–81.0)	36.1 (24.8–49.7)	0.15	0.002	0.006	0.05	0.17	0.28

Abbreviations: LE, lupus erythematosus; PR, primary Raynaud phenomenon; SSc, systemic sclerosis spectrum.

**Table 3:** Comparison of flow-mediated skin fluorescence parameters according to nailfold capillaroscopic pattern.

Parameter	Normal (n = 37)	Nonspecific (n = 40)	Scleroderma-Like (n = 22)	ε^2^	*p* (KW)	FDR *p* (KW)	Partial η^2^	*p* (ANCOVA)	FDR *p* (ANCOVA)	*p* (Trend)	FDR *p* (Trend)
Hypoxia sensitivity (HS)	57.0 (20.8–131.0)	36.2 (18.4–66.7)	17.4 (9.7–72.6)	0.06	0.05	0.10	0.02	0.37	0.62	0.01	0.03
PSD1 (baseline flowmotion power)	72.4 (53.4–129.6)	51.9 (24.2–98.6)	38.0 (9.7–64.1)	0.13	0.002	0.01	0.07	0.02	0.12	0.001	0.005
Myogenic oscillations	14.9 (7.1–32.8)	15.2 (5.0–28.0)	3.9 (1.6–13.9)	0.12	0.002	0.01	0.07	0.04	0.14	0.001	0.005
Endothelial oscillations	22.7 (13.6–42.6)	14.1 (8.7–29.9)	7.7 (3.7–25.3)	0.08	0.02	0.06	0.06	0.07	0.21	0.004	0.01
Neurogenic oscillations	21.0 (11.5–41.2)	18.4 (6.6–44.9)	8.8 (2.6–21.0)	0.08	0.02	0.05	0.04	0.18	0.42	0.008	0.02
PSD2 (reactive flowmotion power)	95.5 (64.9–242.2)	91.6 (47.3–124.6)	93.3 (37.4–152.0)	0.02	0.46	0.66	0.01	0.55	0.62	0.25	0.40
Reactive endothelial oscillations	21.1 (14.3–40.5)	23.8 (14.0–36.9)	28.8 (15.7–51.2)	0.01	0.74	0.78	0.01	0.55	0.62	0.55	0.62
Reactive neurogenic oscillations	11.6 (8.2–21.3)	12.0 (8.2–22.3)	12.2 (5.8–19.7)	0.00	0.88	0.88	0.00	0.97	0.97	0.73	0.77
HR index (%)	14.2 (11.0–16.9)	11.8 (9.4–13.0)	9.6 (7.5–11.8)	0.18	<0.001	0.003	0.15	<0.001	0.007	<0.001	0.004
HR max (%)	21.4 (19.5–24.4)	18.1 (16.1–21.5)	16.5 (14.6–19.9)	0.15	<0.001	0.005	0.14	<0.001	0.007	<0.001	0.004
RHR (%)	32.3 (27.7–40.0)	27.9 (21.7–32.9)	26.4 (20.8–32.4)	0.11	0.004	0.01	0.10	0.008	0.05	0.001	0.005
MR (%)	95.3 (84.1–102.4)	92.9 (83.8–100.4)	79.8 (72.1–98.3)	0.06	0.06	0.11	0.04	0.14	0.37	0.03	0.05
IR max (%)	13.0 (7.3–18.1)	11.6 (6.9–16.6)	12.1 (5.4–17.9)	0.02	0.52	0.66	0.02	0.55	0.62	0.34	0.47
Endothelial (%)	37.6 (20.8–59.1)	34.8 (19.4–48.7)	38.7 (28.1–53.6)	0.01	0.50	0.66	0.01	0.64	0.68	0.89	0.89
Neurogenic (%)	33.7 (20.0–48.5)	38.2 (25.4–50.0)	38.3 (27.5–47.8)	0.01	0.64	0.72	0.01	0.50	0.62	0.37	0.47
Myogenic (%)	20.6 (11.9–38.1)	26.1 (11.8–36.5)	19.6 (10.5–26.1)	0.02	0.39	0.62	0.01	0.55	0.62	0.36	0.47
Reactive endothelial (%)	20.9 (13.6–39.1)	28.2 (16.3–46.6)	45.3 (25.1–57.2)	0.07	0.04	0.10	0.02	0.42	0.62	0.02	0.04
Reactive neurogenic (%)	15.0 (8.5–22.5)	16.7 (11.0–26.4)	15.8 (9.3–26.4)	0.01	0.62	0.72	0.02	0.42	0.62	0.51	0.60
Reactive myogenic (%)	58.2 (35.9–77.6)	53.6 (31.1–68.8)	35.4 (24.4–54.9)	0.06	0.07	0.11	0.02	0.49	0.62	0.03	0.05

**Table 4:** Spearman correlation analysis of age and flow-mediated skin fluorescence parameters.

Parameter	ρ	*p*	FDR *p*	ρ Partial	*p* Partial	FDR *p* Partial
Hypoxia sensitivity (HS)	−0.47	<0.001	<0.001	−0.28	0.006	0.05
PSD1 (baseline flowmotion power)	−0.36	<0.001	0.001	−0.15	0.14	0.36
Myogenic oscillations	−0.36	<0.001	0.001	−0.15	0.15	0.36
Endothelial oscillations	−0.18	0.07	0.14	−0.01	0.92	0.92
Neurogenic oscillations	−0.34	<0.001	0.002	−0.19	0.06	0.28
PSD2 (reactive flowmotion power)	−0.31	0.002	0.005	−0.14	0.17	0.36
Reactive endothelial oscillations	−0.08	0.44	0.49	−0.05	0.65	0.77
Reactive neurogenic oscillations	−0.09	0.37	0.44	−0.05	0.62	0.77
HR index (%)	−0.23	0.02	0.05	−0.10	0.34	0.58
HR max (%)	−0.15	0.15	0.20	−0.08	0.46	0.67
RHR (%)	−0.17	0.09	0.15	−0.03	0.77	0.86
MR (%)	−0.16	0.11	0.16	−0.11	0.28	0.53
IR max (%)	−0.06	0.59	0.59	0.03	0.83	0.87
Endothelial (%)	0.18	0.08	0.14	0.15	0.15	0.36
Neurogenic (%)	−0.07	0.49	0.52	−0.15	0.15	0.36
Myogenic (%)	−0.14	0.16	0.20	−0.06	0.59	0.77
Reactive endothelial (%)	0.39	<0.001	<0.001	0.28	0.007	0.05
Reactive neurogenic (%)	0.26	0.01	0.02	0.09	0.38	0.61
Reactive myogenic (%)	−0.42	<0.001	<0.001	−0.27	0.008	0.05

## Data Availability

Data are available from the corresponding author upon request.

## Supplementary Materials

The following supporting information can be downloaded at https://www.mdpi.com/article/10.3390/jcm15145357/s1: Figure S1: Flowchart of patient selection and inclusion in the study. Table S1: Dictionary of FMSF parameters. Table S2: Multivariable rank-based ANCOVA across clinical groups. Table S3: Sensitivity analyses across different modeling constructs and two approaches. Table S4: Comparison of area under the curve for scleroderma-like capillaroscopic pattern, including model addition of age. Table S5: Comparison of significant age-adjusted capillaroscopy-pattern rank-ANCOVA features, before and after adjustment for CTD diagnosis.
