# Supplementary material for: Flow-Mediated Skin Fluorescence Assessment of Microvascular Function in Connective Tissue Diseases: Associations with Nailfold Capillaroscopic Patterns
Source: J Clin Med. 2026 Jul 9;15(14):5357. doi: 10.3390/jcm15145357 (PMC13410150; doi:10.3390/jcm15145357)
Supplement: Supplementary file 1 [file jcm-15-05357-s001.zip › jcm-4380840-supplementary.pdf]

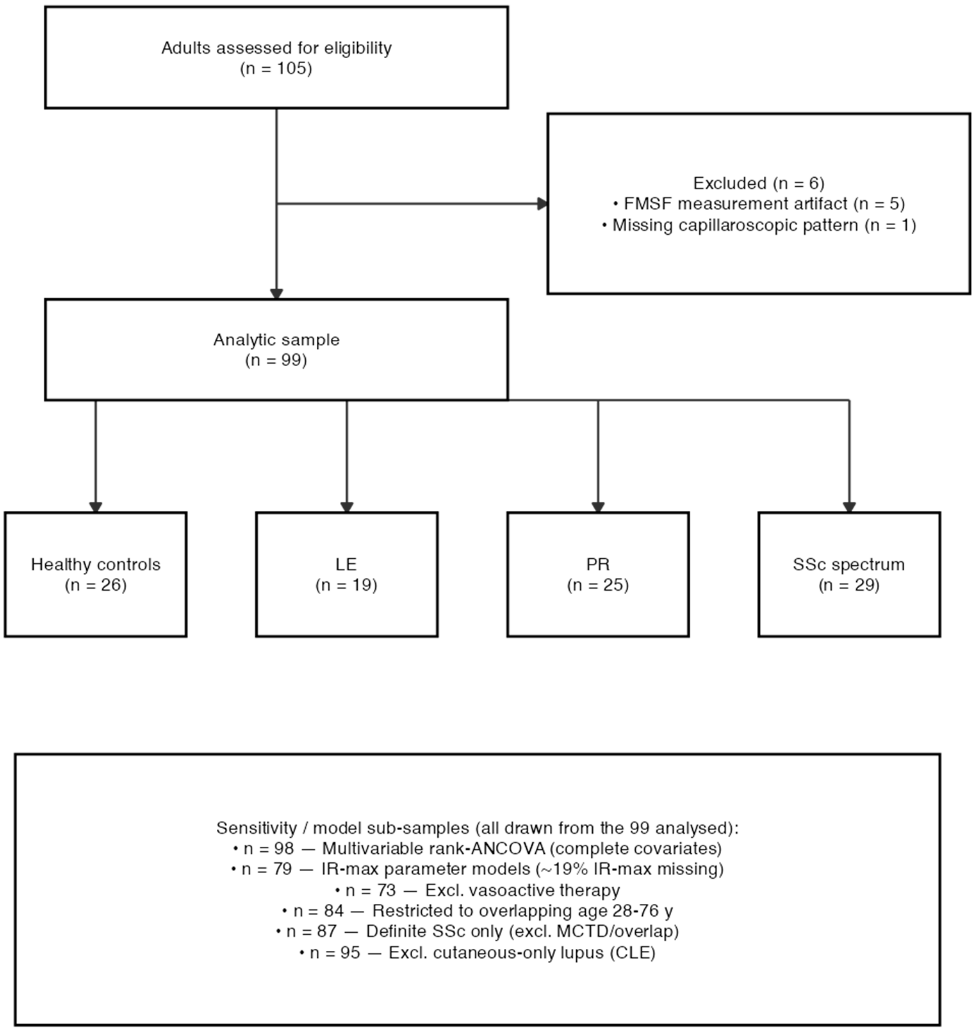

Figure S1. Flowchart of patient selection and inclusion in the study.

Supplementary Table S1. Dictionary of FMSF parameters

Parameter	Domain	Operational definition / interpretation	Expected direction
Hypoxia sensitivity (HS)	Ischemic	A fraction of flowmotion during the reperfusion phase. Measure of the intensity of flowmotion related to myogenic oscillations (0,052-0,15 Hz) recorded during reperfusion. Provides putative characterization of microcirculatory response to transient ischemia	Lower (strongly age-dependent)
PSD1 (baseline flowmotion power)	Baseline	Total spectral power of resting flowmotion oscillations	Lower

PSD2 (reactive flowmotion power)	Reactive	Total spectral power of oscillations after cuff release	Lower
Endothelial oscillations	Baseline	Band ~0.005-0.02 Hz; endothelial activity by LDF analogy	Lower
Neurogenic oscillations	Baseline	Band ~0.02-0.06 Hz; sympathetic activity by LDF analogy	Lower
Myogenic oscillations	Baseline	Band ~0.06-0.15 Hz; vascular smooth-muscle tone by LDF analogy	Lower
Reactive endothelial oscillations	Reactive	Endothelial band power after cuff release	Lower
Reactive neurogenic oscillations	Reactive	Neurogenic band power after cuff release	Lower
HR index (%)	Hyperemic	Hyperemia Response Index. Reflects overall magnitude of the reactive hyperemic response following ischemia.	Lower
HR max (%)	Hyperemic	Maximal hyperemic response after cuff release.	Lower
RHR (%)	Hyperemic	Reactive Hyperemia Response. Reflects endothelial-dependent vascular reactivity, mediated by nitric oxide.	Lower
MR (%)	Hyperemic	Metabolic/return component of the hyperemic response	Lower
IR max (%)	Ischemic	Maximal ischemic-response amplitude during occlusion	Lower
Endothelial (%)	Baseline	Endothelial band as % of total baseline power	Variable
Neurogenic (%)	Baseline	Neurogenic band as % of total baseline power	Variable
Myogenic (%)	Baseline	Myogenic band as % of total baseline power	Variable
Reactive endothelial (%)	Reactive	Endothelial band as % of total reactive power	Variable
Reactive neurogenic (%)	Reactive	Neurogenic band as % of total reactive power	Variable
Reactive myogenic (%)	Reactive	Myogenic band as % of total reactive power	Variable

Supplementary Table S2. Sensitivity analyses across different modeling constructs and two approaches.

Parameter	n	F	df	Partial η^2	p value
Hypoxia sensitivity (HS)	98	7.81	3	0.208	0.002
HR index (%)	98	7.16	3	0.195	0.002
Myogenic oscillations	98	5.31	3	0.152	0.013
PSD1	98	4.84	3	0.140	0.017
PSD2	98	4.02	3	0.119	0.034
HR max (%)	98	3.95	3	0.117	0.034
Endothelial oscillations	98	3.66	3	0.110	0.041
Reactive myogenic (%)	98	2.64	3	0.082	0.128
RHR (%)	98	2.50	3	0.078	0.137

MR (%)	98	2.19	3	0.069	0.181
Reactive endothelial (%)	98	2.07	3	0.065	0.189
Reactive neurogenic (%)	98	1.99	3	0.063	0.192
Neurogenic (%)	98	1.71	3	0.055	0.249
Neurogenic oscillations	98	1.22	3	0.039	0.418
Myogenic (%)	98	0.85	3	0.028	0.599
Reactive neurogenic osc.	98	0.57	3	0.019	0.755
Endothelial (%)	98	0.41	3	0.014	0.790
IR max (%)	79	0.41	3	0.017	0.790
Reactive endothelial osc.	98	0.32	3	0.011	0.814

Descriptive summary of regression for each FMSF parameter on clinical group adjusting for age, sex, BMI, dyslipidemia and hypertension. Partial η^2 corresponds to group effect.

Supplementary Table S3. Sensitivity analyses across different modeling constructs and two approaches

	Kruskal-Wallis test (p value)				Age-adjusted ANCOVA (p value)			
Parameter	No vasoactive (n=73)	Definite SSc (n=87)	SLE only (n=95)	Age 28-76 (n=84)	No vasoactive	Definite SSc	SLE only	Age 28-76
PSD1	0.016	0.005	<0.001	0.004	0.115	0.069	0.025	0.063
Endothelial oscillations	0.022	0.011	0.006	0.016	0.115	0.069	0.025	0.063
Myogenic oscillations	0.016	0.007	<0.001	0.007	0.115	0.139	0.025	0.180
Hypoxia sensitivity (HS)	0.022	0.002	<0.001	0.005	0.289	0.139	0.041	0.180
HR index (%)	0.128	0.082	0.012	0.025	0.248	0.306	0.041	0.166
PSD2	0.129	0.016	0.017	0.061	0.470	0.208	0.229	0.447
HR max (%)	0.537	0.076	0.141	0.074	0.621	0.228	0.267	0.180
Reactive neurogenic (%)	0.053	0.033	0.025	0.034	0.248	0.459	0.229	0.212
MR (%)	0.053	0.745	0.048	0.321	0.115	0.760	0.120	0.447
RHR (%)	0.170	0.401	0.090	0.278	0.470	0.760	0.267	0.502
Neurogenic oscillations	0.128	0.070	0.025	0.061	0.621	0.459	0.385	0.448
Reactive myogenic (%)	0.136	0.023	0.008	0.025	0.621	0.593	0.267	0.447
Endothelial (%)	0.132	0.204	0.181	0.374	0.339	0.306	0.267	0.447
Reactive endothelial (%)	0.537	0.101	0.032	0.106	0.862	0.760	0.495	0.736
Myogenic (%)	0.405	0.440	0.299	0.421	0.476	0.722	0.385	0.646
Neurogenic (%)	0.625	0.322	0.471	0.558	0.585	0.306	0.267	0.502
Reactive endothelial osc.	0.436	0.355	0.608	0.604	0.533	0.459	0.680	0.646
IR max (%)	0.537	0.480	0.736	0.746	0.585	0.593	0.816	0.809
Reactive neurogenic osc.	0.852	0.592	0.726	0.788	0.862	0.722	0.816	0.809

Supplementary Table S4. Comparison of area under the curve for scleroderma-like capillaroscopic pattern, including model addition of age.

Parameter	AUC (95% CI)	p value	AUC-Age	p value vs age
HR index (%)	0.74 (0.62-0.85)	0.009	0.83	0.039
Myogenic oscillations	0.73 (0.62-0.85)	0.009	0.80	0.248
PSD1	0.72 (0.60-0.84)	0.010	0.80	0.303
Neurogenic oscillations	0.69 (0.55-0.83)	0.033	0.76	0.905
HR max (%)	0.68 (0.56-0.81)	0.034	0.80	0.266
Reactive endothelial (%)	0.67 (0.53-0.80)	0.049	0.77	0.931
MR (%)	0.67 (0.51-0.82)	0.049	0.79	0.450
Endothelial oscillations	0.66 (0.52-0.80)	0.057	0.78	0.481
Reactive myogenic (%)	0.64 (0.51-0.77)	0.087	0.77	0.820
Hypoxia sensitivity (HS)	0.64 (0.50-0.78)	0.087	0.76	0.894
RHR (%)	0.62 (0.48-0.75)	0.176	0.79	0.369
Myogenic (%)	0.59 (0.46-0.73)	0.284	0.77	0.661
Endothelial (%)	0.56 (0.43-0.69)	0.574	0.75	0.146
Reactive endothelial osc.	0.55 (0.41-0.70)	0.592	0.76	0.932
PSD2	0.54 (0.40-0.69)	0.672	0.76	0.502
Neurogenic (%)	0.54 (0.41-0.67)	0.696	0.77	0.718
Reactive neurogenic osc.	0.53 (0.39-0.68)	0.696	0.76	0.533
IR max (%)	0.53 (0.36-0.70)	0.755	0.78	1.000
Reactive neurogenic (%)	0.50 (0.36-0.64)	0.966	0.75	0.632

Summary of discriminatory performance of models for scleroderma-like versus normal/nonspecific pattern (n event / sample = 22 / 99). Additionally, the model AUC for a logistic model with age and specific FMSF parameters is reported (defined as "AUC-Age"); p value vs. age corresponds to DeLong p value approach comparison to age model alone (age-only AUC = 0.76).

Supplementary Table S5. Comparison of significant age-adjusted capillaroscopy-pattern rank-ANCOVA features, before and after adjustment for CTD diagnosis.

Parameter	Age-adjusted p value	Age + CTD-adjusted p value
HR index (%)	0.007	0.075
HR max (%)	0.007	0.065
RHR (%)	0.049	0.075

Descriptive summary of rank-ANCOVA for each FMSF parameter that was significant after age adjustment, with modeling for capillaroscopic patterns. However, after additional correction for CTD subgroup, we did not observe significant FMSF parameters. Age-adjusted p value presented is after FDR correction.
